# Supplementary material for: Differences in Cell-Intrinsic Inflammatory Programs of Yolk Sac and Bone Marrow Macrophages
Source: Cells. 2021 Dec 17;10(12):3564. doi: 10.3390/cells10123564 (PMC8699930; doi:10.3390/cells10123564)
Supplement: Supplementary file 1 [file cells-10-03564-s001.zip › cells-1496987 SM.pdf]

Supplemental Figures

Sara Elhag, Christopher Stremmel, et al. Differences in cell-intrinsic inflammatory programs of yolk sac and bone marrow macrophages

Supplemental Figure S1

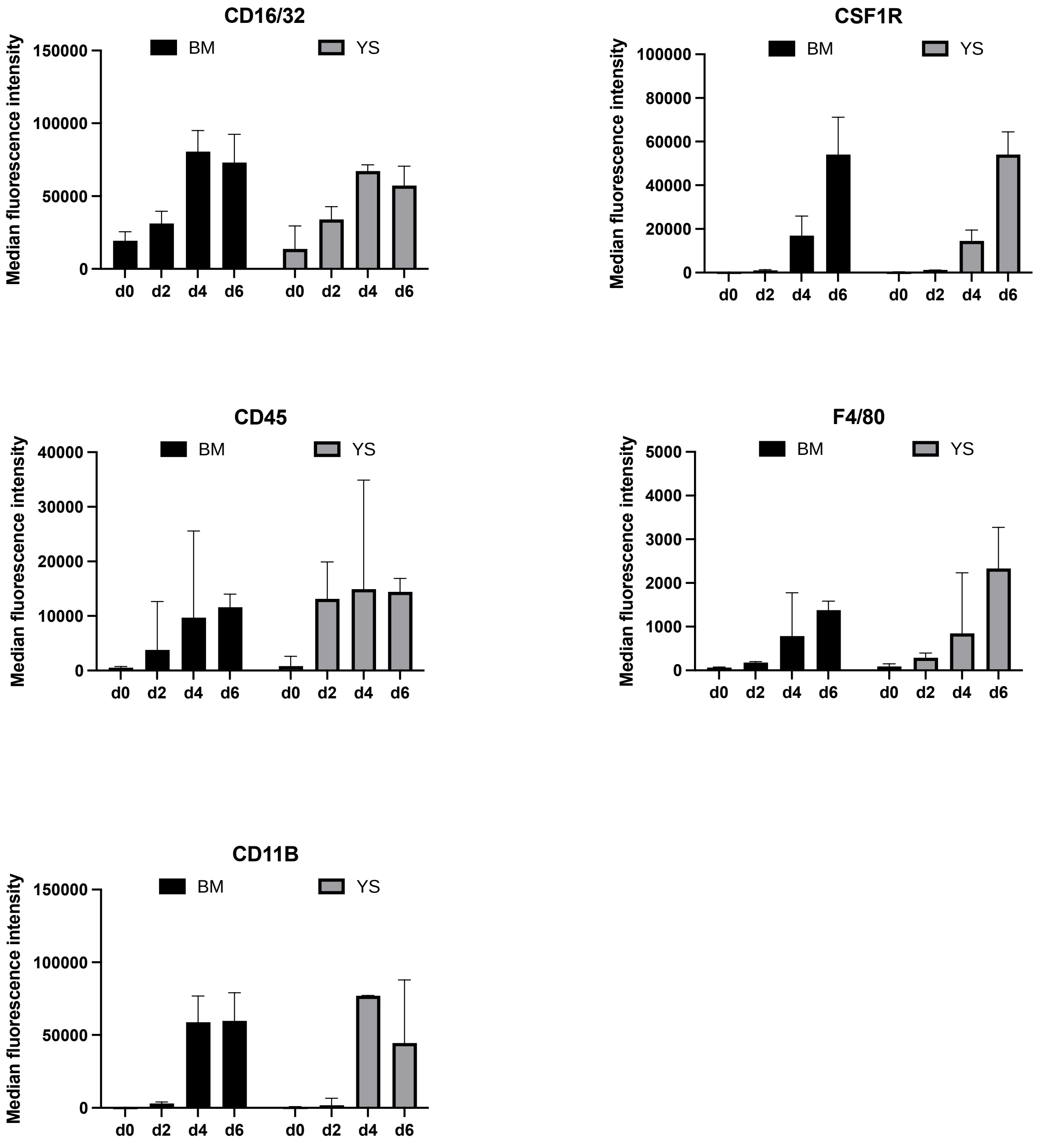

Figure S1: Median fluorescence intensities of immune cell markers. Flow cytometry of BM and YS Hoxb8 macrophages at different days of differentiation. Day (d)0 represents undifferentiated state. Median fluorescence intensity + interquartile range is indicated. N=3 per each timepoint.

Supplemental Figure S2

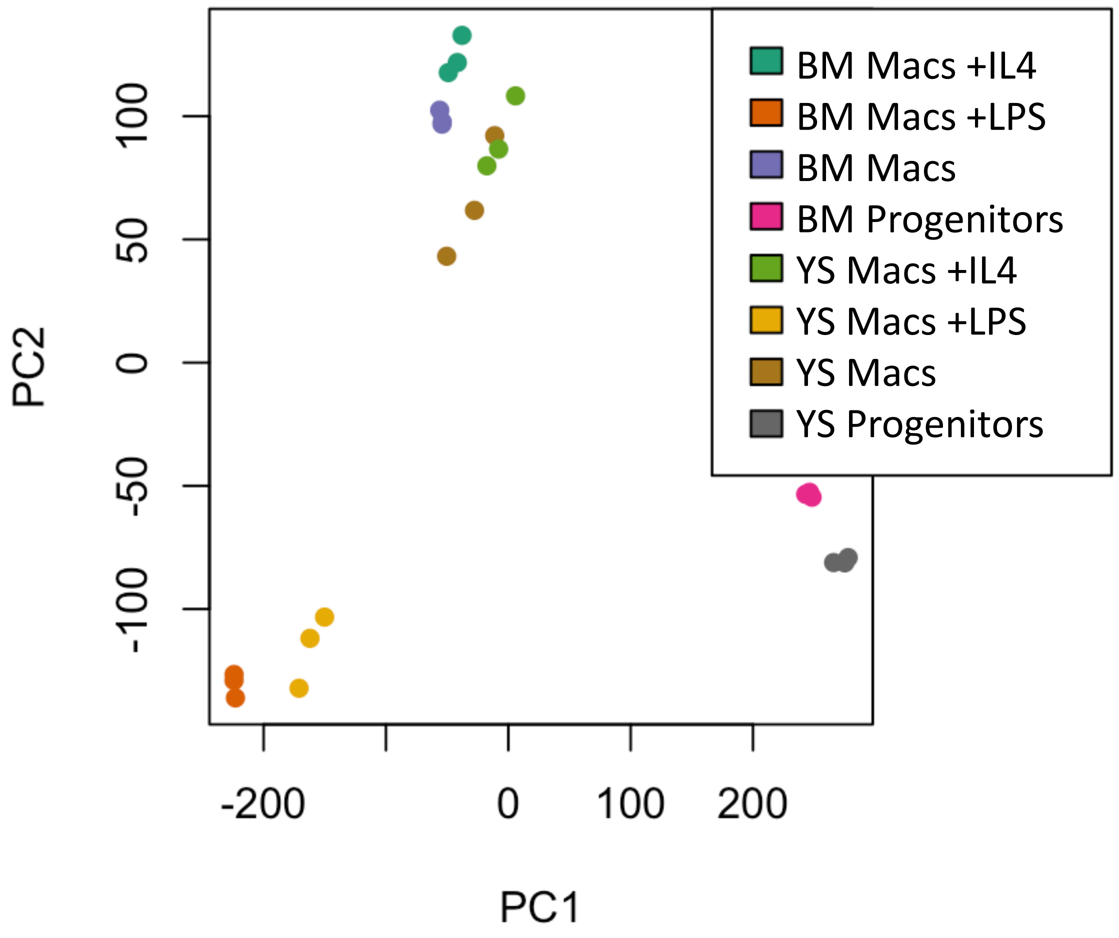

Figure S2: Principal component analysis for RNA expression profiles of YS and BM Hoxb8 progenitors and differentiated macrophages either unstimulated or stimulated with IL4 or LPS as indicated (n=3 independent experiments).

Supplemental Figure S3

A) Venn diagram

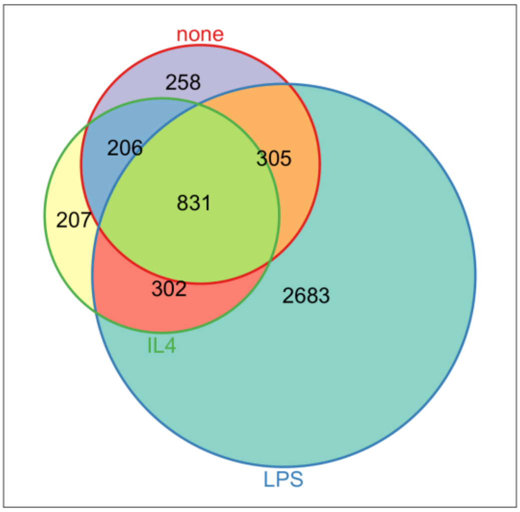

B) Top10 differentially expressed genes per condition

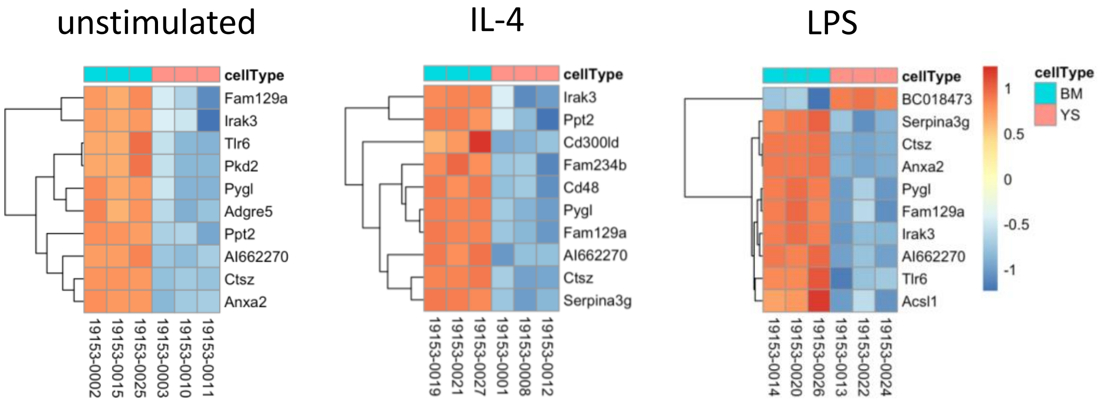

Figure S3: Differential gene expression of YS compared to BM Hoxb8 macrophages in response to IL-4 and LPS stimulation. A) Venn diagram of the differentially expressed genes related to the transcriptome analysis in main figure 3. Cutoff FDR < 0.1. B) Heatmap display of top10 differentially regulated genes for YS versus BM in unstimulated macrophages as well as IL-4 and LPS stimulation.

Supplemental Figure S4

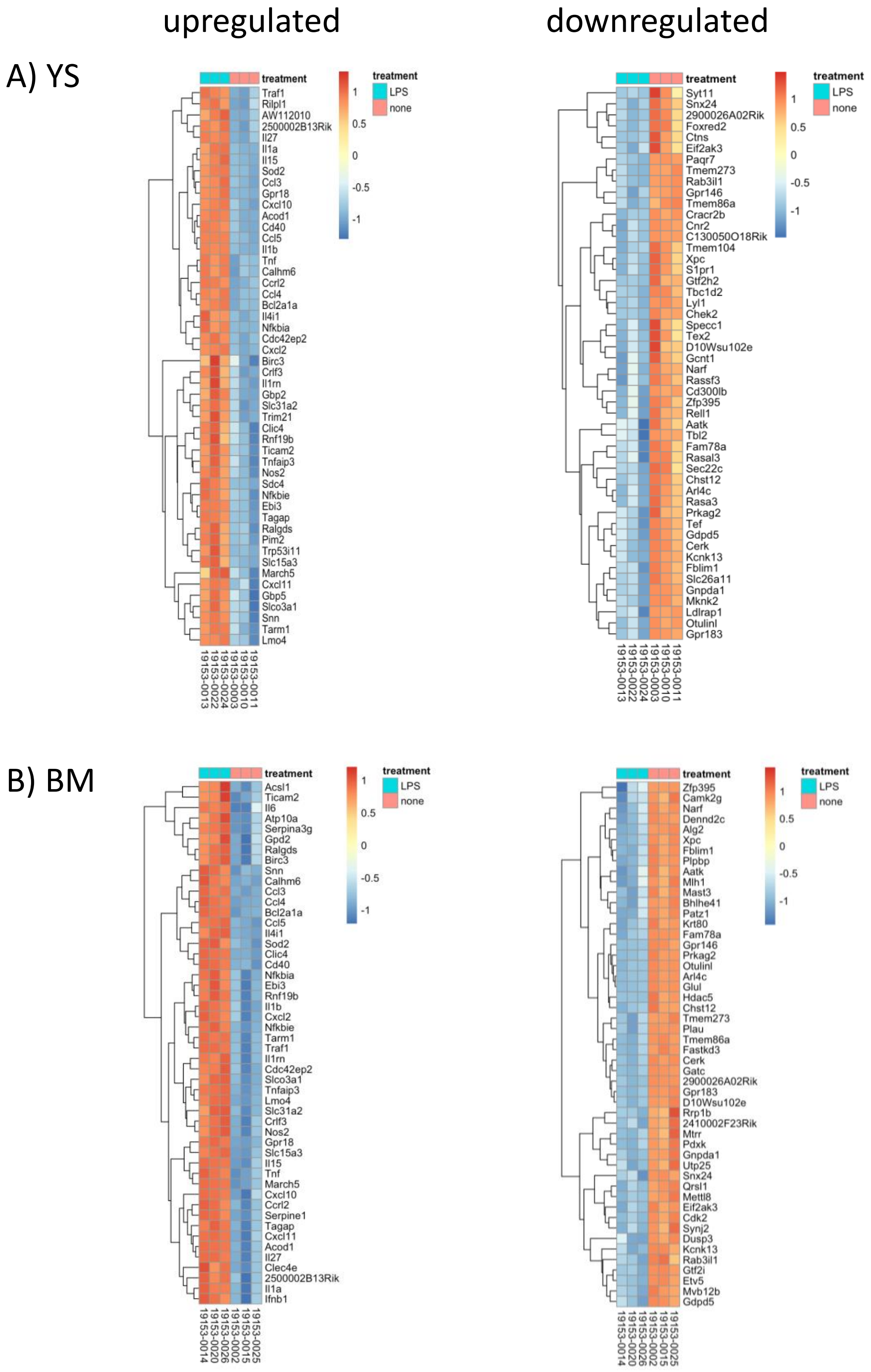

Figure S4: LPS-induced changes in gene expression in YS (A) and BM (B) Hoxb8 macrophages. Heatmap display of top50 upregulated (left panels) and downregulated (right panels) genes.

Supplemental Figure S5

GSEA
Hallmark gene sets

Pathway	Gene ranks	NES	pval	padj
HALLMARK_OXIDATIVE_PHOSPHORYLATION		2.99	4.5e-03	1.2e-02
HALLMARK_G2M_CHECKPOINT		2.44	5.1e-03	1.2e-02
HALLMARK_IL2_STAT5_SIGNALING		-1.89	1.3e-03	7.8e-03
HALLMARK_INFLAMMATORY_RESPONSE		-2.17	1.3e-03	7.8e-03
HALLMARK_INTERFERON_ALPHA_RESPONSE		-2.28	1.4e-03	7.8e-03
HALLMARK_INTERFERON_GAMMA_RESPONSE		-2.45	1.2e-03	7.8e-03

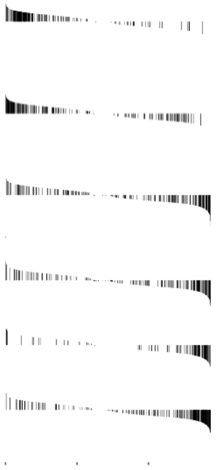

GO gene sets

Pathway	Gene ranks	NES	pval	padj
_MITOCHONDRIAL_MEMBRANE_PROTEIN_COMPLEX		2.85	3.5e-03	4.0e-02
GO_MITOCHONDRIAL_PROTEIN_COMPLEX		2.81	3.6e-03	4.0e-02
GO_RESPIRATORY_CHAIN		2.69	3.2e-03	4.0e-02
GO_ORGANELLAR_RIBOSOME		2.57	3.0e-03	4.0e-02
)RIAL_RESPIRATORY_CHAIN_COMPLEX_ASSEMBLY		2.55	3.2e-03	4.0e-02
GO_RESPONSE_TO_INTERFERON_GAMMA		-2.30	1.3e-03	3.5e-02
IVE_REGULATION_OF_INNATE_IMMUNE_RESPONSE		-2.31	1.4e-03	3.5e-02
GO_DEFENSE_RESPONSE_TO_VIRUS		-2.35	1.2e-03	3.5e-02
RFERON_GAMMA_MEDIATED_SIGNALING_PATHWAY		-2.38	1.4e-03	3.5e-02
)_CELLULAR_RESPONSE_TO_INTERFERON_GAMMA		-2.41	1.3e-03	3.5e-02

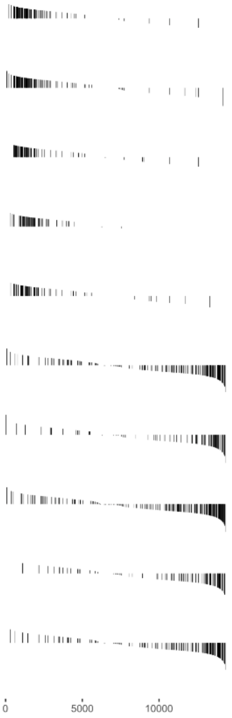

Figure S5: Gene Set (GSEA) and Gene Ontology (GO) term enrichment analysis of Hoxb8 YS and BM macrophages.

Supplemental Figure S6

A) OCR

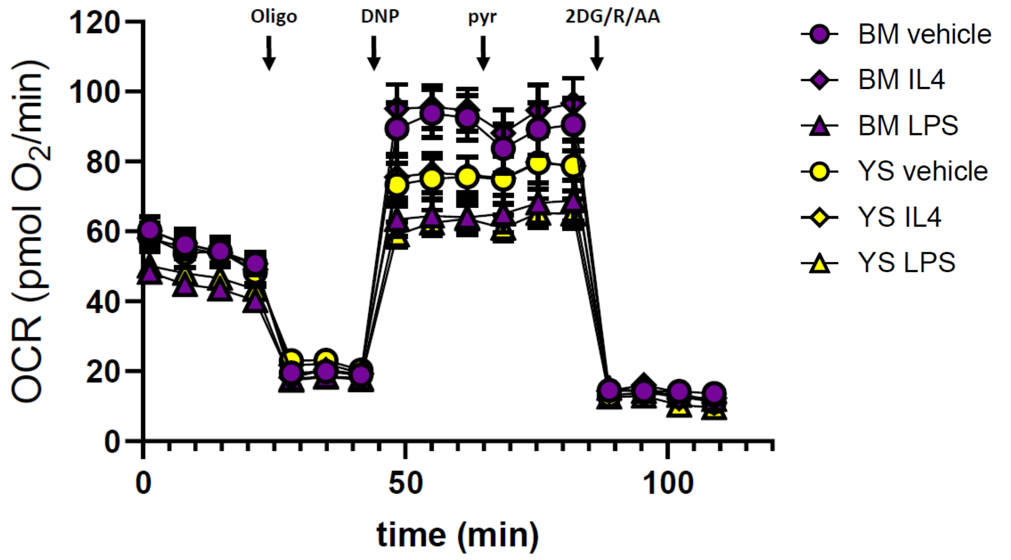

B) ECAR

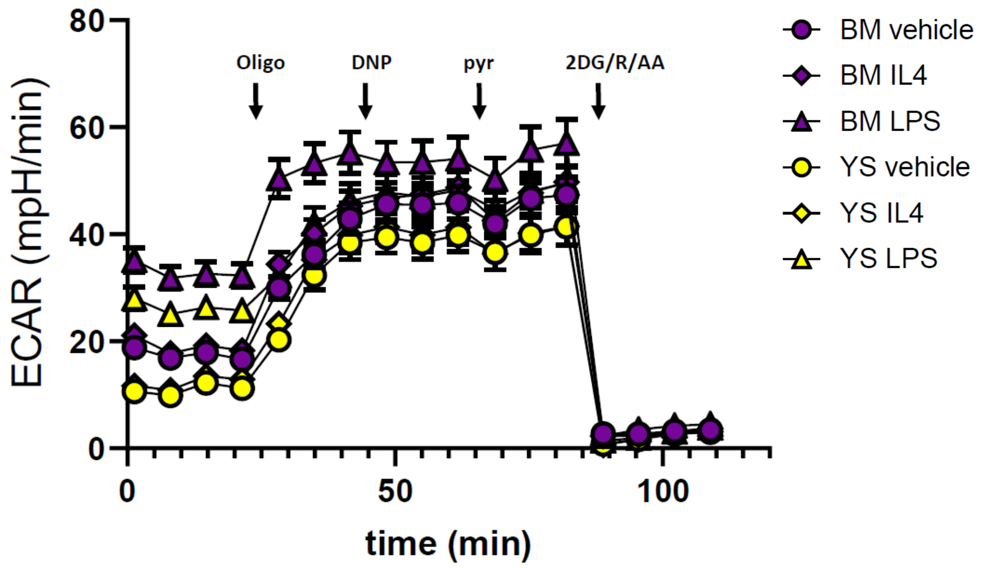

Figure S6: Measurements of oxygen consumption rate (OCR, panel A) and extracellular acidification rate (ECAR, panel B). Time laps traces for BM and YS Hoxb8 macrophages stimulated with LPS or IL4 4h prior to Seahorse XF96 run. Data are the mean +/- SEM of 26-29 wells per condition measured on three independent days.

Supplemental Figure S7

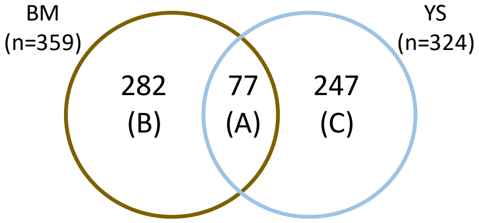

A) Overlap (n=77)

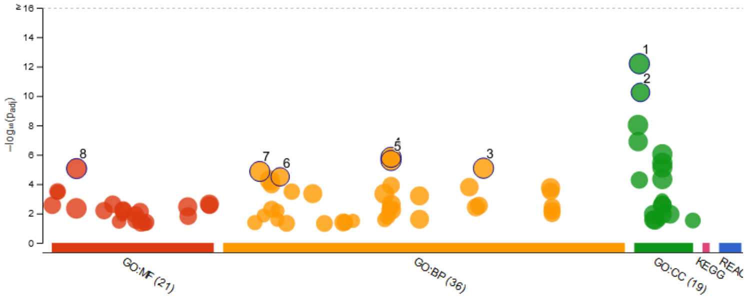

ID	Sour...	Term ID	Term Name	p_{adj} (query_1)
1	GO:CC	GO:0005737	cytoplasm	6.399×10^{-13}
2	GO:CC	GO:0005829	cytosol	5.704×10^{-11}
3	GO:BP	GO:0071704	organic substance metabolic process	8.261×10^{-6}
4	GO:BP	GO:0044238	primary metabolic process	1.495×10^{-6}
5	GO:BP	GO:0044237	cellular metabolic process	2.464×10^{-6}
6	GO:BP	GO:0010033	response to organic substance	3.180×10^{-5}
7	GO:BP	GO:0006807	nitrogen compound metabolic process	1.370×10^{-5}
8	GO:MF	GO:0005515	protein binding	8.772×10^{-6}

B) BM (n=359)

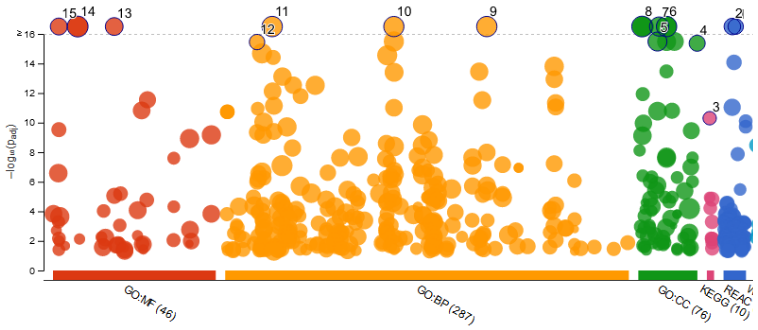

ID	Source	Term ID	Term Name	p_{adj} (query_1)
1	REAC	REAC:R-MMU-6798695	Neutrophil degranulation	1.768×10^{-17}
2	REAC	REAC:R-MMU-168249	Innate Immune System	3.043×10^{-17}
3	KEGG	KEGG:03040	Spliceosome	5.031×10^{-11}
4	GO:CC	GO:1990904	ribonucleoprotein complex	4.263×10^{-16}
5	GO:CC	GO:0031974	membrane-enclosed lumen	3.289×10^{-16}
6	GO:CC	GO:0043231	intracellular membrane-bounded organelle	1.899×10^{-27}
7	GO:CC	GO:0032991	protein-containing complex	9.837×10^{-25}
8	GO:CC	GO:0005622	intracellular anatomical structure	2.485×10^{-41}
9	GO:BP	GO:0071704	organic substance metabolic process	4.963×10^{-18}
10	GO:BP	GO:0044237	cellular metabolic process	2.110×10^{-18}
11	GO:BP	GO:0008152	metabolic process	1.023×10^{-19}
12	GO:BP	GO:0006397	mRNA processing	3.540×10^{-16}
13	GO:MF	GO:0019899	enzyme binding	6.494×10^{-17}
14	GO:MF	GO:0005488	binding	9.217×10^{-18}
15	GO:MF	GO:0003723	RNA binding	4.933×10^{-24}

C) YS (n=324)

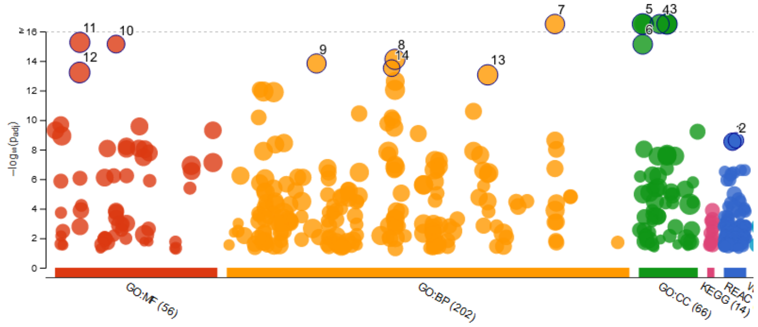

ID	Source	Term ID	Term Name	p_{adj} (query_1)
1	REAC	REAC:R-MMU-168256	Immune System	2.932×10^{-9}
2	REAC	REAC:R-MMU-6798...	Neutrophil degranulation	2.388×10^{-9}
3	GO:CC	GO:0043231	intracellular membrane-bounded organelle	5.852×10^{-24}
4	GO:CC	GO:0032991	protein-containing complex	1.635×10^{-23}
5	GO:CC	GO:0005622	intracellular anatomical structure	3.541×10^{-32}
6	GO:CC	GO:0005634	nucleus	7.516×10^{-16}
7	GO:BP	GO:1901564	organonitrogen compound metabolic process	2.701×10^{-18}
8	GO:BP	GO:0044237	cellular metabolic process	7.762×10^{-15}
9	GO:BP	GO:0019538	protein metabolic process	1.478×10^{-14}
10	GO:MF	GO:0019899	enzyme binding	7.199×10^{-16}
11	GO:MF	GO:0005515	protein binding	5.501×10^{-16}
12	GO:MF	GO:0005488	binding	6.041×10^{-14}
13	GO:BP	GO:0071704	organic substance metabolic process	8.704×10^{-14}
14	GO:BP	GO:0043603	cellular amide metabolic process	3.045×10^{-14}

Figure S7: g:profiler enrichment analysis indicates LPS-induced changes in protein abundance and related pathways for proteins altered in both cell types (A), and for BM (B) and YS (C) Hoxb8 macrophages. $p< 0.05$.

Supplemental Figure S8

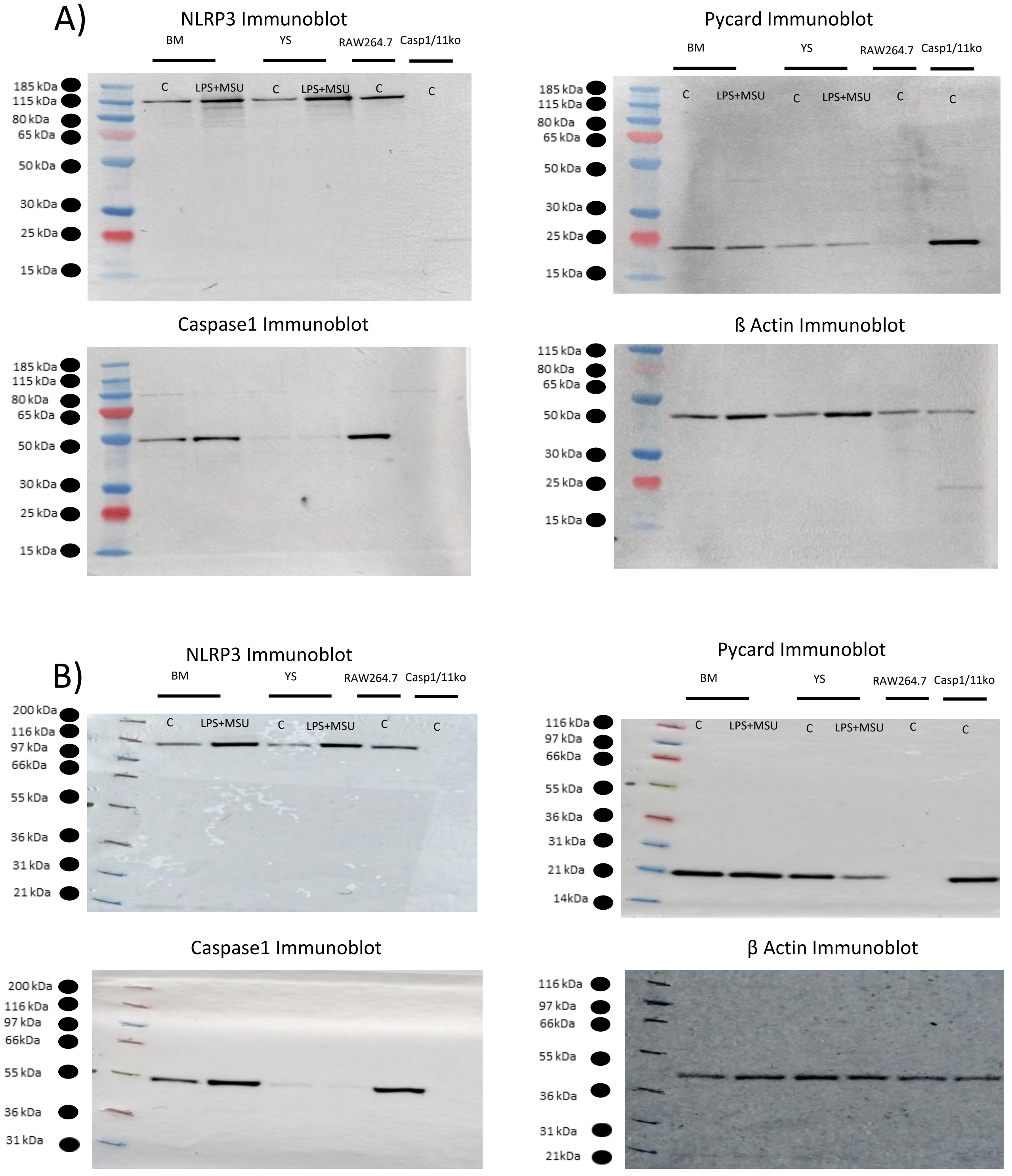

Supplemental Figure S8 (continued)

c)

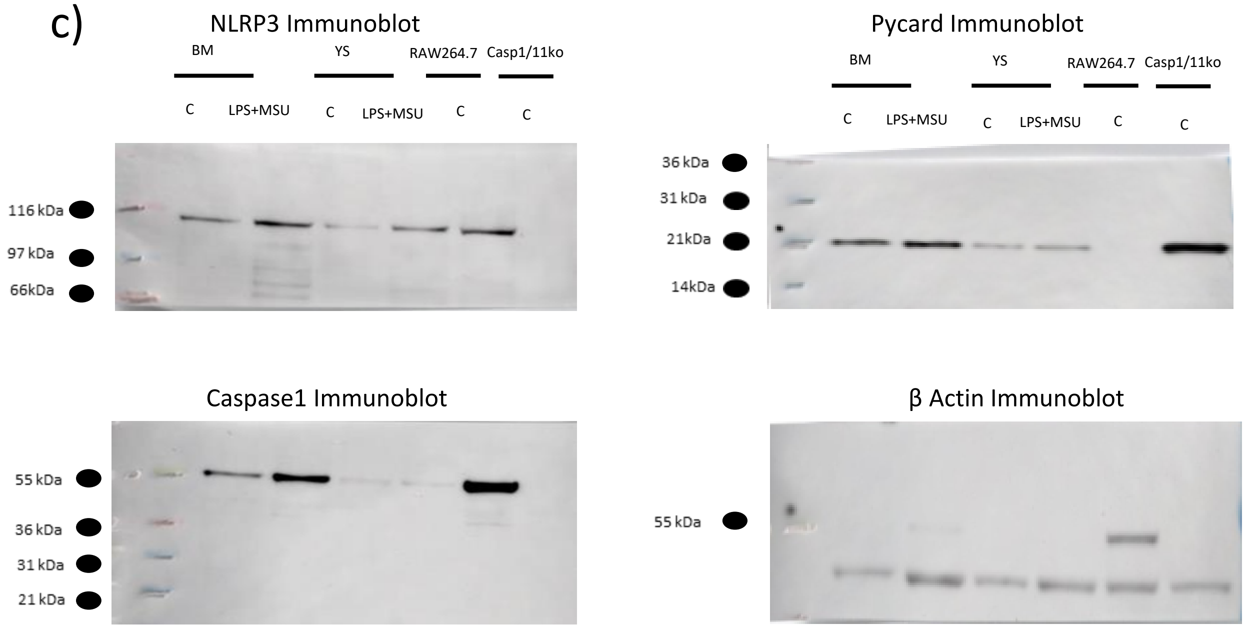

Figure S8: Full images of immunoblots related to figures 4f-g. (A-C) NLRP3 (118kDa), Caspase1 (50kDa), Pycard (21.6 kDa) and β-actin (42 kDa) expression in BM and YS Hoxb8 macrophages after 3 hours of LPS priming and 24 hours of stimulation with MSU crystals, compared to control (C). RAW264.7 cells and Hoxb8 macrophages from Casp1/11-knockout mice were used as control.

Supplemental Figure S9

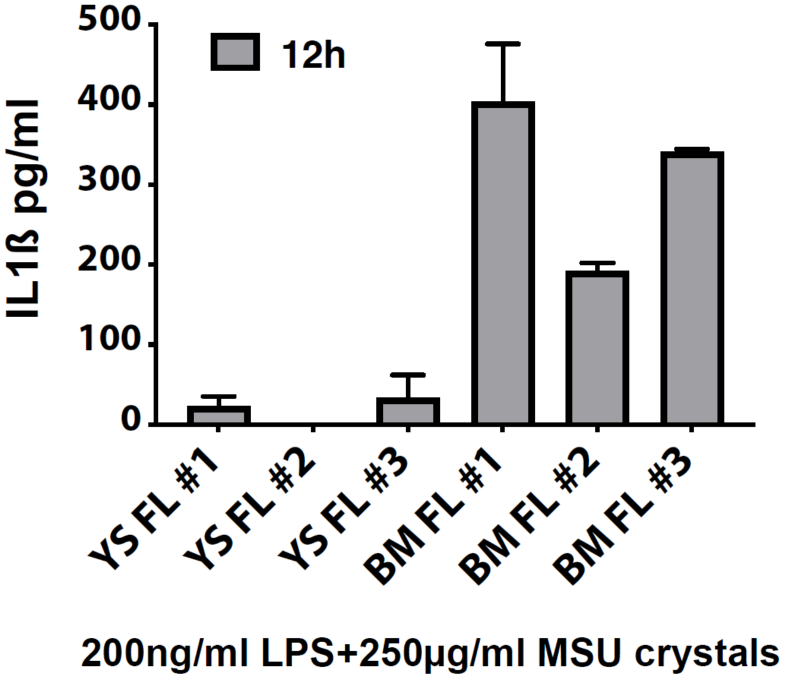

Figure S9: IL1β expression analysis by ELISA in the supernatant of LPS-primed crystal-stimulated (12h MSU) YS and BM HoxB8 FL macrophages.
